# Relationship Between Emergency Medical Services Response Time and Bystander Intervention in Patients With Out‐of‐Hospital Cardiac Arrest

**DOI:** 10.1161/JAHA.117.007568

**Published:** 2018-04-27

**Authors:** Yoshikazu Goto, Akira Funada, Yumiko Goto

**Affiliations:** ^1^ Department of Emergency and Critical Care Medicine Kanazawa University Hospital Kanazawa Japan; ^2^ Department of Cardiology Yawata Medical Center Komatsu Japan

**Keywords:** cardiopulmonary resuscitation, epidemiology, heart arrest, resuscitation, Cardiopulmonary Resuscitation and Emergency Cardiac Care

## Abstract

**Background:**

The response time of emergency medical services (EMS) is an important determinant of survival after out‐of‐hospital cardiac arrest. We sought to identify upper limits of EMS response times and bystander interventions associated with neurologically intact survival.

**Methods and Results:**

We analyzed the records of 553 426 patients with out‐of‐hospital cardiac arrest in a Japanese registry between 2010 and 2014. The primary study end point was 1‐month neurologically intact survival (Cerebral Performance Category scale 1 or 2). Increased EMS response time was associated with significantly decreased adjusted odds of 1‐month neurologically intact survival (adjusted odds ratio [aOR] for each 1‐minute increase, 0.89; 95% confidence interval [CI], 0.89–0.90), although this relationship was modified by bystander interventions. The bystander interventions and the ranges of EMS response times that were associated with increased adjusted 1‐month neurologically intact survival were as follows: bystander defibrillation, from ≤2 minutes (aOR, 3.10 [95% CI, 1.25–7.31]) to 13 minutes (aOR, 5.55 [95% CI, 2.66–11.2]); bystander conventional cardiopulmonary resuscitation, from 3 minutes (aOR 1.48 [95% CI, 1.02–2.12]) to 11 minutes (aOR 2.41 [95% CI, 1.61–3.56]); and bystander chest‐compression‐only cardiopulmonary resuscitation, from ≤2 minutes (aOR 1.57 [95% CI, 1.01–2.25]) to 11 minutes (aOR 1.92 [95% CI, 1.45–2.56]). However, the increase in neurologically intact survival of those receiving bystander interventions became statistically insignificant compared with no bystander interventions when the EMS response time was outside these ranges.

**Conclusions:**

The upper limits of the EMS response times associated with improved 1‐month neurologically intact survival were 13 minutes when bystanders provided defibrillation (typically with cardiopulmonary resuscitation) and 11 minutes when bystanders provided cardiopulmonary resuscitation without defibrillation.

**Clinical Perspective**

**What Is New?**

- Data from the All‐Japan Utstein Registry indicated that bystander interventions were associated with significantly increased 1‐month neurologically intact survival, based on the following 3 scenarios: bystander defibrillation, with emergency medical services (EMS) response times ranging from ≤2 to 13 minutes; conventional cardiopulmonary resuscitation, with EMS response times ranging from 3 to 11 minutes; and chest‐compression‐only cardiopulmonary resuscitation, with EMS response times ranging from ≤2 to 11 minutes.
- When EMS response time exceeded these ranges, no significant differences remained between the outcomes of patients who received bystander defibrillations and those who did not.

**What Are the Clinical Implications?**

- The maximum EMS response time associated with improved neurologically intact survival was 13 minutes when bystanders provided defibrillation (typically with cardiopulmonary resuscitation), and 11 minutes when bystanders provided cardiopulmonary resuscitation without defibrillation.
- Beyond these time ranges, 1‐month neurologically intact survival did not improve as a result of bystander intervention.
- Therefore, appropriate initiatives should be adopted to reduce EMS response time, and initiatives that aim to improve out‐of‐hospital cardiac arrest outcomes should also focus on factors besides EMS response time that may influence survival.

## Introduction

Following a cardiac arrest, early bystander cardiopulmonary resuscitation (CPR) and defibrillation are both vital components in the chain of survival.^1^, ^2^ However, the proportion of patients who receive bystander CPR, following an out‐of‐hospital cardiac arrest (OHCA), is still relatively low in most countries.^3^ Fortunately, bystander CPR rates in some countries have recently increased to ≈50%.^4^, ^5^, ^6^, ^7^, ^8^, ^9^ In an analysis of OHCA, Hasselqvist‐Ax and colleagues^6^ reported survival that was more than twice as high among patients who received bystander CPR compared with those who received no CPR before the arrival of emergency medical services (EMS) providers. In other studies, bystander interventions (bystander CPR and defibrillation) were associated with increased 1‐month neurologically intact survival,^8^ and lower rate of neurological compromise or nursing home admission.^9^ Recently, Rajan et al^10^ reported that among patients with OHCA who received bystander CPR, absolute 30‐day survival declined substantially in association with increased EMS response times, and survival of those with and without bystander CPR no longer differed significantly once EMS response time exceeded 13 minutes. Therefore, we hypothesized that EMS response time might be associated with 1‐month neurologically intact survival and that the maximum time taken for EMS arrival, to maintain the benefits of bystander intervention, might be <13 minutes.

Using Japanese registry data from adult patients with OHCA, we aimed to (1) examine the relationship between EMS response times and bystander interventions and 1‐month neurologically intact survival, and (2) explore the upper limit of beneficial EMS response times linked with 3 types of bystander interventions: defibrillation, conventional CPR, and chest‐compression‐only CPR.

## Methods

### Study Design and Setting

The data, analytic methods, and study materials will not be made available to other researchers for purposes of reproducing the results or replicating the procedure. This nationwide, population‐based, observational study included all adult patients (age ≥18 years) with an episode of OHCA who received attempted resuscitation by EMS personnel in Japan between January 1, 2010 and December 31, 2014. This study was approved by the ethical committee of Kanazawa University. The requirement for written informed consent was waived as the study involved the second use of anonymous data.

Japan has nearly 127 million residents, in an area of 378 000 km^2^; approximately two thirds of this area is uninhabited, mountainous terrain.^11^ The Fire and Disaster Management Agency (FDMA) of Japan supervises the nationwide EMS system, while local fire stations operate the local EMS systems. As of 2014, there were 752 fire departments, with 5028 ambulance teams.^12^ During the study period, all the EMS providers performed CPR according to the Japanese CPR guidelines.^13^, ^14^ In addition to CPR, emergency lifesaving technicians who are EMS providers perform several resuscitation techniques, including use of automated external defibrillators (AEDs), insertion of airway adjuncts, insertion of peripheral intravenous catheters, and administration of Ringer's lactate solution.^12^, ^13^ Only specially trained emergency lifesaving technicians, receiving instruction from an online physician, are permitted to insert a tracheal tube and administer intravenous epinephrine in the field.^13^ Additionally, EMS personnel in Japan are legally prohibited from terminating resuscitation in the field, except in specific situations such as decapitation, incineration, decomposition, rigor mortis, or dependent cyanosis. As a result, most patients with OHCA undergo CPR by EMS providers and are subsequently transported to the hospital.^13^, ^15^ The appropriate duration of on‐scene CPR by EMS personnel before transport to a hospital has not been predetermined.

### Data Collection and Quality Control

In 2005, the FDMA launched an ongoing, prospective, population‐based, observational study involving all patients with OHCA who received resuscitation by EMS personnel in Japan.^12^, ^15^ Since 2005, with the cooperation of the physician in charge, EMS personnel at each center recorded data from patients with OHCA using an Utstein‐style template.^16^, ^17^ The data are transferred to local, individual fire stations and subsequently integrated into the registry system on the FDMA database server. The database software program automatically checks data for consistency, verified by the FDMA. If a data form is incomplete, the FDMA returns it to the respective fire station and the form is completed. All the data are transferred and stored in the nationwide database developed by the FDMA for public use. The FDMA granted permission to access the database and provided the anonymous data for our analysis.

The main variables included in the data set were as follows: sex, age, cause of arrest, initially identified cardiac rhythm, bystander witness status, category of the witness (family member, a layperson other than family, EMS personnel, etc), presence and maneuvers of bystander CPR, time of collapse recognition, time of emergency call receipt, time of vehicle arrival at the scene, time of EMS initiation of CPR, 1‐month survival, and neurological outcomes 1 month after a cardiac arrest. EMS time at the scene is not currently recorded in the database. The cause of an arrest was presumed to be cardiac unless evidence suggested an external cause such as trauma, hanging, drowning, drug overdose, asphyxia, respiratory disease, cerebrovascular disease, malignant tumors, or other noncardiac causes. The physicians in charge and EMS personnel determined the origin of the arrest. The time data are recorded electronically using a recording device. Neurological outcomes were defined using the Cerebral Performance Category scale (category 1: good cerebral performance, category 2: moderate cerebral disability, category 3: severe cerebral disability, category 4: coma or vegetative state, and category 5: death).^16^ The Cerebral Performance Category categorization was determined by the physician in charge. We defined EMS response time as the time from the call receipt by an EMS center to the arrival of an ambulance at the site of an OHCA.

### Study End Points

The primary study end point was neurologically intact survival, defined as a Cerebral Performance Category of 1 or 2, at 1 month. The secondary end point was 1‐month survival, following an OHCA.

### Statistical Analysis

To determine the association between EMS response times and bystander interventions with 1‐month outcomes from OHCA, we divided patients into 4 cohorts based on the type of bystander intervention: bystander defibrillation (whether or not CPR was performed with the defibrillation), bystander conventional CPR (chest compressions with rescue breaths), bystander chest‐compression‐only CPR, and absence of bystander intervention. Continuous variables were expressed as medians and 25th to 75th percentiles or as mean and SD, whereas categorical variables were expressed as percentages. We used the Kruskal–Wallis test followed by the Dunn post‐hoc test to analyze the continuous variables. Chi‐square tests and univariate logistic regression analyses were performed for categorical variables to compare the characteristics and outcomes between the cohorts. Multivariate logistic regression analyses, including 10 prehospital variables, were performed to evaluate the association between EMS response time and 1‐month outcomes for all the eligible patients. The potential prehospital confounders for the analytic model were selected based on biological plausibility and data from previous studies. The 10 selected variables included age, sex, bystander‐witnessed arrest (yes or no), initial shockable rhythm (yes or no), presumed cardiac cause (yes or no), prehospital AED administration by EMS personnel (yes or no), use of advanced airway management (yes or no), epinephrine administration (yes or no), type of bystander intervention (bystander defibrillation, bystander conventional CPR, or bystander chest‐compression‐only CPR), and EMS response time.

We calculated the crude and adjusted OHCA 1‐month outcomes by EMS response times and bystander interventions. For each EMS response time, univariate and multivariate logistic regression analyses were performed to compare the 1‐month outcomes among the 4 cohorts sorted on the basis of bystander interventions. Model‐adjusted rates of 1‐month outcomes were computed against the reference value of the absence of bystander CPR after calculating the adjusted odds ratios (ORs) of bystander interventions for 1‐month outcomes.

All statistical analyses were performed using the JMP statistical package, version 13 (SAS Institute Inc, Cary, NC). All the reported tests were 2‐tailed, and statistical significance was established at *P*<0.05.

## Results

**Figure 1:**
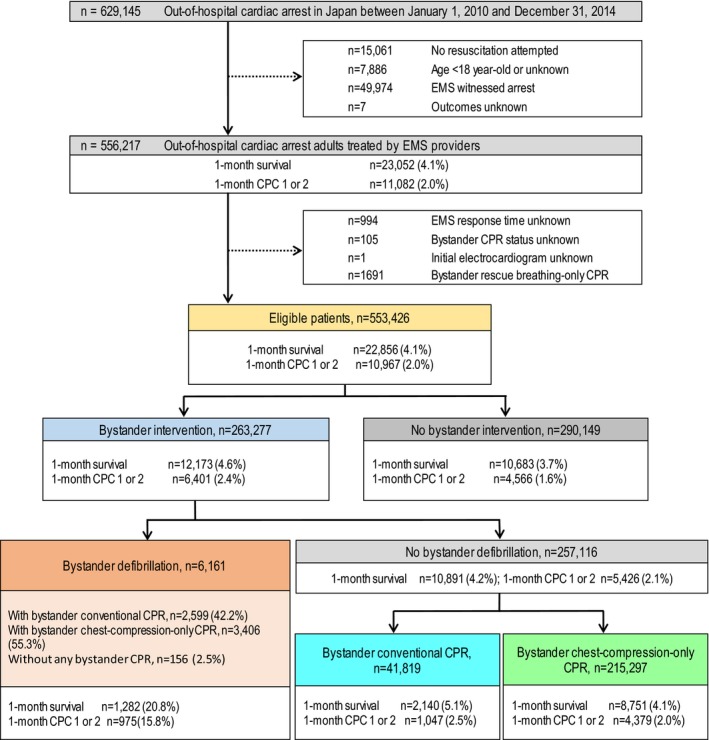
Study inclusion flowchart. CPC indicates Cerebral Performance Category scale; CPR, cardiopulmonary resuscitation; EMS, emergency medical services.

From 2010 to 2014, details of attempted resuscitation in Japan of 629 145 patients with OHCA were documented in the database. Figure 1 shows the inclusion and exclusion criteria for subjects in the present study. Among 556 217 adult patients (aged ≥18 years) who were treated by EMS personnel after an EMS unwitnessed cardiac arrest, patients without information on EMS response time (n=994), bystander CPR status (n=105), and initial cardiac rhythm (n=1), and those with bystander rescue breathing–only CPR (n=1691) were excluded. Finally, 553 426 patients (88.0% of registered patients) with OHCA were eligible for enrollment in this study. Patients who received bystander defibrillation (n=6161) consisted of 3 subgroups: with bystander conventional CPR (n=2599, 42.2%), with bystander chest‐compression‐only CPR (n=3406, 55.3%), and without any bystander CPR (n=156, 2.5%).

Table 1 shows the baseline characteristics of the study patients. Patients who received bystander defibrillation were significantly younger than those in the other cohorts (*P*<0.001). In the cohort that received bystander defibrillation (as compared with other cohorts), the proportions of males, those with witnessed arrest, with an initial shockable rhythm, with a presumed cardiac cause, with prehospital AED administration by EMS personnel, and with epinephrine administration were significantly higher (all *P*<0.001). The rate of the use of advanced airway management in those with bystander defibrillation was significantly lower than that in the other cohorts (*P*<0.001). The EMS response time in those without bystander intervention was significantly shorter than that in the other 3 cohorts (*P*<0.001). The 1‐month outcomes in the 3 cohorts that received any of the bystander interventions were all significantly higher than outcomes in the cohort that received no bystander intervention (all *P*<0.001).

**Table 1:** Baseline Characteristics of the Participants According to Bystander Status

Characteristic	No Bystander Intervention	Bystander Intervention			*P* Value
	n=290 149 (52.4%)	Bystander Defibrillation	Conventional CPR	Chest‐Compression‐Only CPR	
		n=6161 (1.1%)	n=41 819 (7.6%)	n=215 297 (38.9%)	
Year					<0.001
2010	59 103 (20.4)	1076 (17.4)	10 336 (24.7)	38 136 (17.7)	
2011	61 054 (21.0)	1162 (18.8)	9486 (22.7)	40 819 (19.0)	
2012	59 635 (20.6)	1477 (24.0)	8264 (19.8)	43 563 (20.2)	
2013	55 142 (19.0)	1174 (19.1)	6685 (16.0)	45 053 (20.9)	
2014	55 215 (19.0)	1272 (20.7)	7048 (16.8)	47 726 (22.2)	
Age, y, median (25–75%)	77 (65–85)	74 (60–85)	82 (70–88)	80 (69–87)	<0.001
Male	174 581 (60.2)	4099 (66.5)	19 676 (47.0)	115 969 (53.9)	<0.001
Witnessed arrest	100 310 (34.6)	3776 (61.3)	18 194 (43.5)	75 732 (35.2)	<0.001
Initial shockable rhythm	18 283 (6.3)	1617 (26.3)	3173 (7.6)	16 110 (7.5)	<0.001
Presumed cardiac cause	165 008 (56.9)	4802 (77.9)	25 246 (60.4)	129 707 (60.3)	<0.001
Prehospital AED administration by EMS personnel	26 192 (9.0)	2128 (34.5)	4217 (10.1)	20 892 (9.7)	<0.001
Use of advanced airway management	114 869 (39.6)	2293 (37.2)	18 279 (43.7)	94 888 (44.1)	<0.001
Epinephrine administration	41 297 (14.2)	1315 (21.3)	6983 (16.7)	32 801 (15.2)	<0.001
EMS response time, min					<0.001
Median (25–75%)	7 (5–9)	7 (5–10)	7 (6–9)	7 (6–9)	
Mean±SD	7.7±3.7	8.1±4.3	8.1±4.0	8.0±3.8	
1‐mo survival, % (25–75%)	3.7 (3.6–3.8)	20.8 (19.8–21.8)	5.1 (4.9–5.3)	4.0 (4.0–4.2)	<0.001
1‐mo neurologically intact survival (CPC 1 or 2), % (25–75%)	1.6 (1.5–1.6)	15.8 (14.9–16.8)	2.5 (2.4–2.7)	2.0 (2.0–2.1)	<0.001

Values are reported as n (%) unless indicated otherwise. AED indicates automated external defibrillator; CPC, Cerebral Performance Category scale; CPR, cardiopulmonary resuscitation; EMS, emergency medical services.

Table 2 shows the adjusted ORs of the prehospital variables for 1‐month outcomes in the multivariate logistic regression models. Any bystander intervention was associated with significantly increased 1‐month survival and neurological outcome when compared with no bystander intervention. Increased EMS response times were associated with significantly decreased 1‐month survival (adjusted OR for each 1‐minute increase, 0.91; 95% confidence interval [CI], 0.90–0.91) and 1‐month neurologically intact survival (adjusted OR for each 1‐minute increase, 0.89; 95% CI, 0.89–0.90). Conversely, reduction of the EMS response time by 1 minute was associated with significantly improved odds of 1‐month survival (adjusted OR, 1.10; 95% CI, 1.10–1.11) and 1‐month neurologically intact survival (adjusted OR, 1.12; 95% CI, 1.11–1.13).

**Table 2:** Adjusted ORs of Prehospital Variables for 1‐Month Outcomes

Variables	Adjusted OR (95% CI)	
	1‐Mo Survival	1‐Mo CPC 1 or 2
Age^a^	0.98 (0.98–0.98)	0.97 (0.97–0.97)
Male (vs female)	1.04 (1.01–1.07)	1.13 (1.08–1.18)
Witnessed arrest (vs unwitnessed arrest)	3.76 (3.64–3.88)	4.00 (3.81–4.20)
Initial shockable rhythm (vs initial nonshockable rhythm)	3.69 (3.48–3.91)	4.10 (3.78–4.45)
Presumed cardiac cause (vs noncardiac causes)	0.84 (0.82–0.87)	1.51 (1.43–1.59)
Prehospital AED administration by EMS personnel (vs no use of EMS AED)	2.25 (2.13–2.38)	2.80 (2.58–3.04)
Use of advanced airway management (vs no use of airway management)	0.72 (0.70–0.75)	0.41 (0.39–0.43)
Epinephrine administration (vs no use of epinephrine)	0.81 (0.78–0.84)	0.39 (0.37–0.42)
Type of bystander intervention (vs no bystander intervention)		
Bystander defibrillation	3.36 (3.12–3.63)	4.72 (4.31–5.17)
Bystander conventional CPR	1.37 (1.30–1.44)	1.54 (1.43–1.66)
Bystander chest‐compression‐only CPR	1.14 (1.10–1.17)	1.32 (1.26–1.38)
EMS response time^a^	0.91 (0.90–0.91)	0.89 (0.89–0.90)

AED indicates automated external defibrillator; CI, confidence interval; CPC, Cerebral Performance Category scale; CPR, cardiopulmonary resuscitation; EMS, emergency medical services; ORs, odds ratio.

a Adjusted ORs are reported for 1‐y or 1‐min increments.

**Figure 2:**
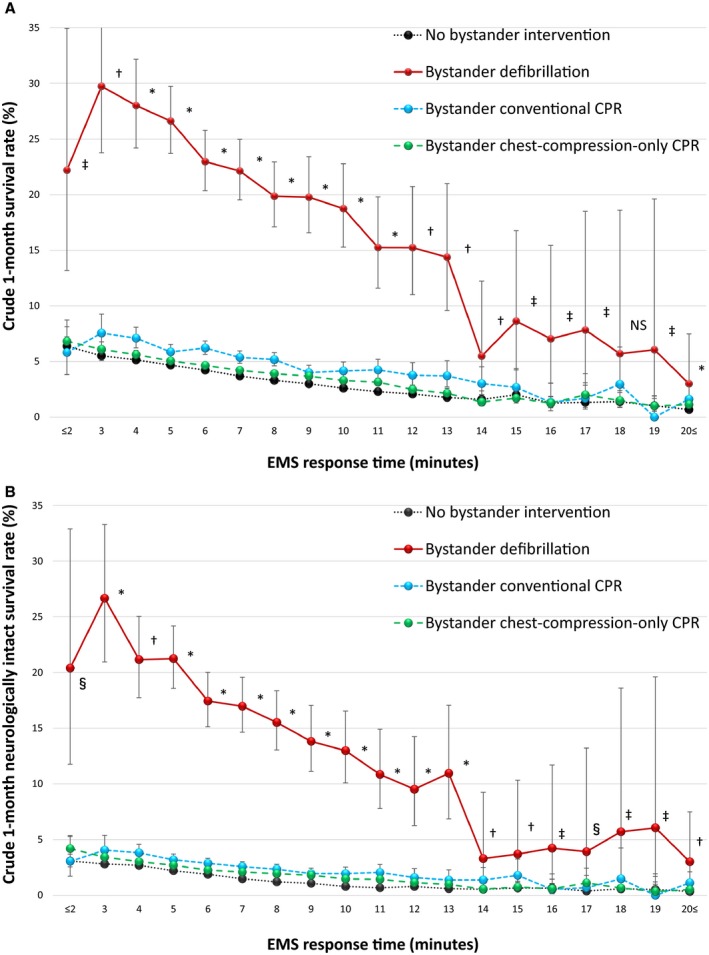
Crude 1‐month outcomes by EMS response time. A, Crude 1‐month survival rate. B, Crude 1‐month neurologically intact survival rate. CPR indicates cardiopulmonary resuscitation; EMS, emergency medical services; NS, not significant among the 4 cohorts. *P<0.05; bystander defibrillation, bystander conventional CPR, and bystander chest‐compression‐only CPR vs no bystander intervention. ^†^ *P*<0.01; bystander defibrillation and bystander conventional CPR vs no bystander intervention. ^‡^ *P*<0.05; bystander defibrillation vs no bystander intervention. ^§^ *P*<0.05; bystander defibrillation and bystander chest‐compression‐only CPR vs no bystander intervention.

Crude (unadjusted) 1‐month outcomes are depicted in Figure 2A and 2B (ie, 1‐month survival and 1‐month neurologically intact survival rates) with EMS response time, in the 4 cohorts of patients receiving some or no bystander interventions. At all EMS response times except at 18 minutes, crude 1‐month outcomes associated with any bystander interventions were significantly higher than outcomes of those who received no bystander intervention.

**Figure 3:**
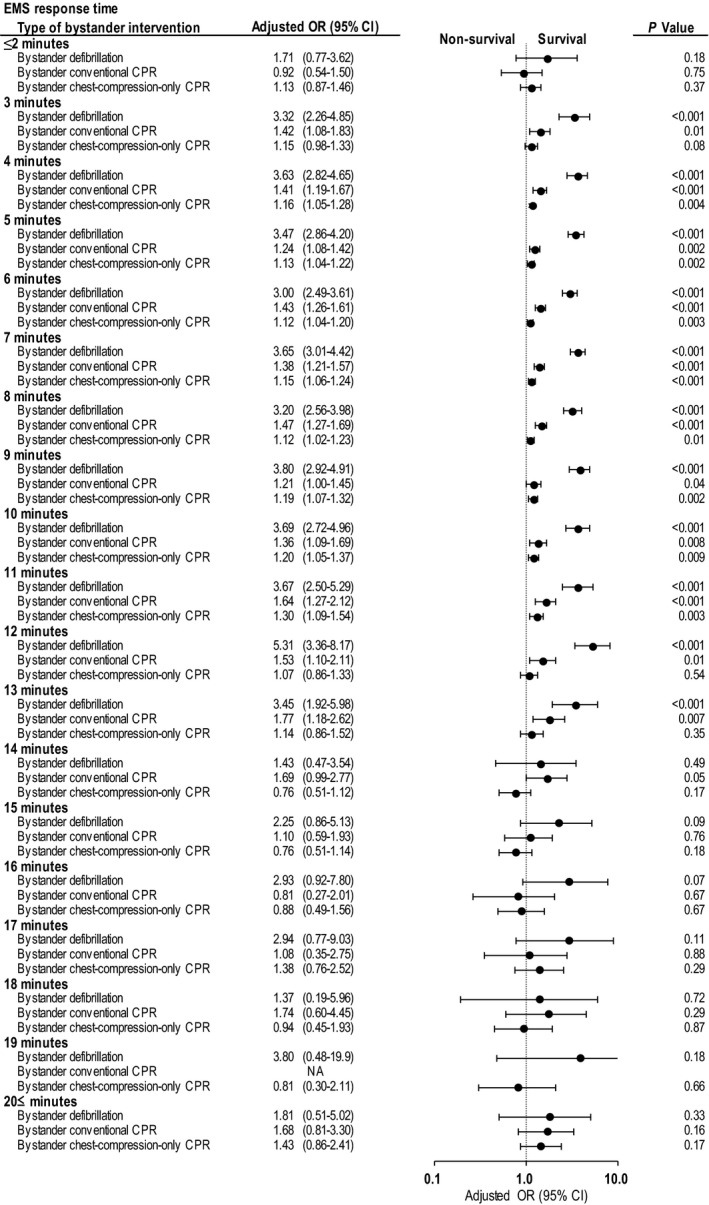
Adjusted OR* of bystander interventions for 1‐month survival by EMS response time. CI indicates confidence interval; CPR, cardiopulmonary resuscitation; EMS, emergency medical services; NA, not available; OR, odds ratio. *Compared with no bystander intervention, adjusted ORs were calculated using a predefined set of 8 potential confounders: age, sex, witnessed arrest, initial shockable rhythm, cardiac cause, automated external defibrillator administration by EMS personnel, use of advanced airway management, and epinephrine administration.

Adjusted 1‐month survival is depicted in Figure 3 with Forest plots, organized by EMS response times and bystander interventions in the 4 cohorts of patients receiving some or no bystander interventions. The bystander interventions and the ranges of EMS response times that were associated with significantly increased 1‐month survival are as follows: bystander defibrillation with EMS response times ranging from 3 to 13 minutes; bystander conventional CPR with EMS response times ranging from 3 to 13 minutes; and bystander chest‐compression‐only CPR with EMS response times ranging from 4 to 11 minutes. When the EMS response times exceeded these ranges, significant differences ceased to exist between patients with bystander interventions and those without.

**Figure 4:**
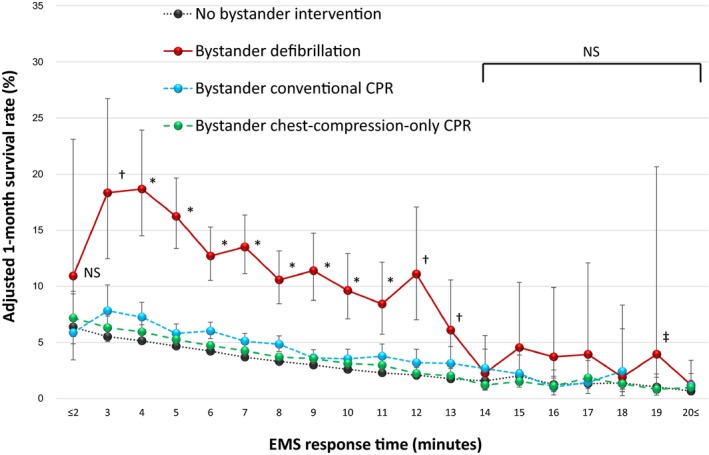
Adjusted 1‐month survival rates by EMS response time. CPR indicates cardiopulmonary resuscitation; EMS, emergency medical services; NS, not significant among the 4 cohorts. *P<0.05; bystander defibrillation, bystander conventional CPR, and bystander chest‐compression‐only CPR vs no bystander intervention. ^†^ *P*<0.01; bystander defibrillation and bystander conventional CPR vs no bystander intervention. ^‡^At 19 minutes, the rate of bystander conventional CPR was not calculated because of the lack of adjusted odds ratios.

Adjusted 1‐month survival rates are depicted in Figure 4, organized by EMS response time, with separate survival curves for each cohort of bystander intervention. The difference in 1‐month survival rates of those patients receiving bystander defibrillation, bystander conventional CPR, and bystander chest‐compression‐only CPR when compared with those who received no bystander interventions became statistically insignificant once the EMS response time was 12 to 14 minutes or longer. These survival rates and EMS response times at which bystander interventions (versus no bystander intervention) do not improve survival are as follows: for bystander defibrillation and EMS response time of 14 minutes (2.3% [95% CI, 0.7–5.6%] versus 1.6% [95% CI, 1.3–2.0%], *P*=0.49), bystander conventional CPR and EMS response time of 14 minutes (2.7% [95% CI, 1.6–4.4%] versus 1.6% [95% CI, 1.3–2.0%], *P*=0.05), and bystander chest‐compression‐only CPR and EMS response time of 12 minutes (2.2% [95% CI, 1.8–2.8%] versus 2.1% [95% CI, 1.81–2.4%], *P*=0.54).

**Figure 5:**
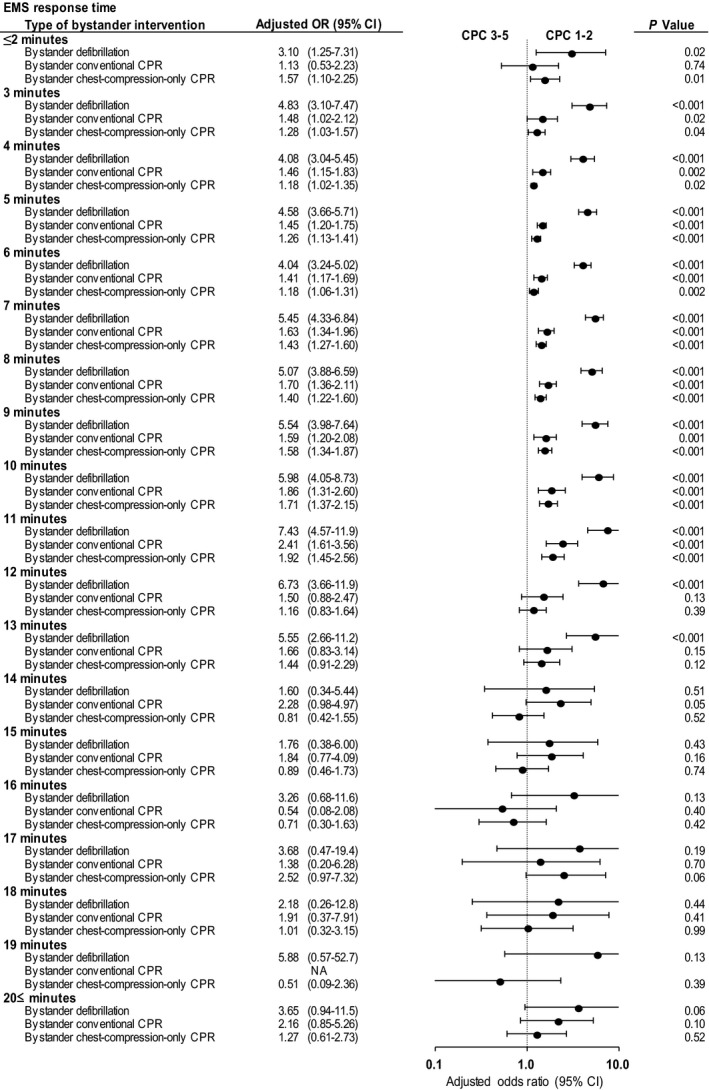
Adjusted ORs* for 1‐month neurologically intact survival by EMS response time. CI indicates confidence interval; CPC, Cerebral Performance Category scale; CPR, cardiopulmonary resuscitation; EMS, emergency medical services; NA, not available; OR, odds ratio. *Compared with no bystander intervention, adjusted ORs were calculated using a predefined set of potential 8 confounders: age, sex, witnessed arrest, initial shockable rhythm, cardiac cause, automated external defibrillator administration by EMS personnel, use of advanced airway management, and epinephrine administration.

Adjusted 1‐month neurologically intact survival is depicted in Figure 5 with a Forest plot organized according to EMS response times (in minutes) and bystander interventions. Bystander interventions were associated with significantly increased 1‐month neurologically intact survival at the following ranges of EMS response times: bystander defibrillation with EMS response times ranging from ≤2 to 13 minutes; bystander conventional CPR with EMS response times ranging from 3 to 11 minutes; and bystander chest‐compression‐only CPR with EMS response times ranging from ≤2 to 11 minutes. When the EMS response time exceeded these ranges, significant differences ceased to exist between the outcomes of patients who received bystander interventions or CPR and those who received no bystander interventions.

**Figure 6:**
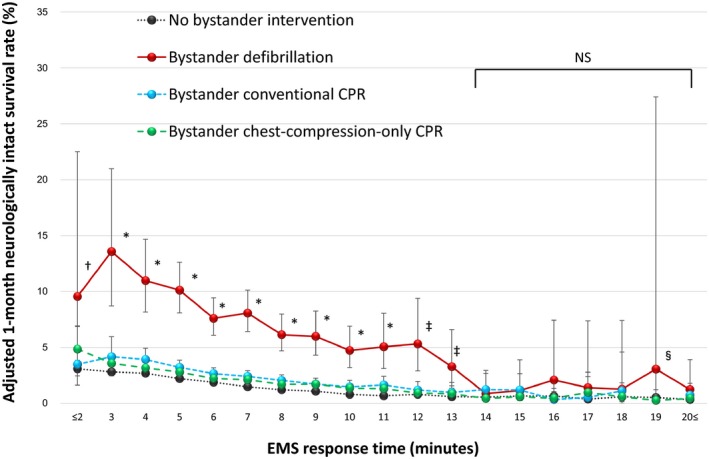
Adjusted 1‐month neurologically intact survival rates by EMS response time. CPR indicates cardiopulmonary resuscitation; EMS, emergency medical services; NS, not significant among the 4 cohorts. *P<0.05; bystander defibrillation, bystander conventional CPR, and bystander chest‐compression‐only CPR vs no bystander intervention. ^†^ *P*<0.01; bystander defibrillation and bystander chest‐compression‐only CPR vs no bystander intervention. ^‡^ *P*<0.001; bystander defibrillation vs no bystander intervention. ^§^At 19 minutes, the rate of bystander conventional CPR was not calculated because of the lack of adjusted odds ratios.

The adjusted 1‐month neurologically intact survival rates are depicted in Figure 6, shown at different EMS response times with separate curves for each type of bystander intervention. The effects of bystander interventions on 1‐month neurologically intact survival gradually decreased with increasing EMS response times. The improvement in 1‐month neurologically intact survival associated with bystander defibrillation, bystander conventional CPR, and bystander chest‐compression‐only CPR became statistically insignificant compared with those who received no bystander interventions once the EMS response time was 12 to 14 minutes or longer. These neurologically intact survival rates and EMS response times at which bystander interventions (versus no bystander intervention) do not improve neurologically intact survival are as follows: for bystander defibrillation and EMS response time of 14 minutes (0.9% [95% CI, 0.2–2.9%] versus 0.5% [95% CI, 0.4–0.8%], *P*=0.51), bystander conventional CPR and EMS response time of 12 minutes (1.2% [95% CI, 0.7–2.0%] versus 0.8% [95% CI, 0.6–1.0%], *P*=0.13), and bystander chest‐compression‐only CPR and EMS response time of 12 minutes (0.9% [95% CI, 0.7–1.3%] versus 0.8% [95% CI, 0.6–1.0%], *P*=0.39).

**Figure 7:**
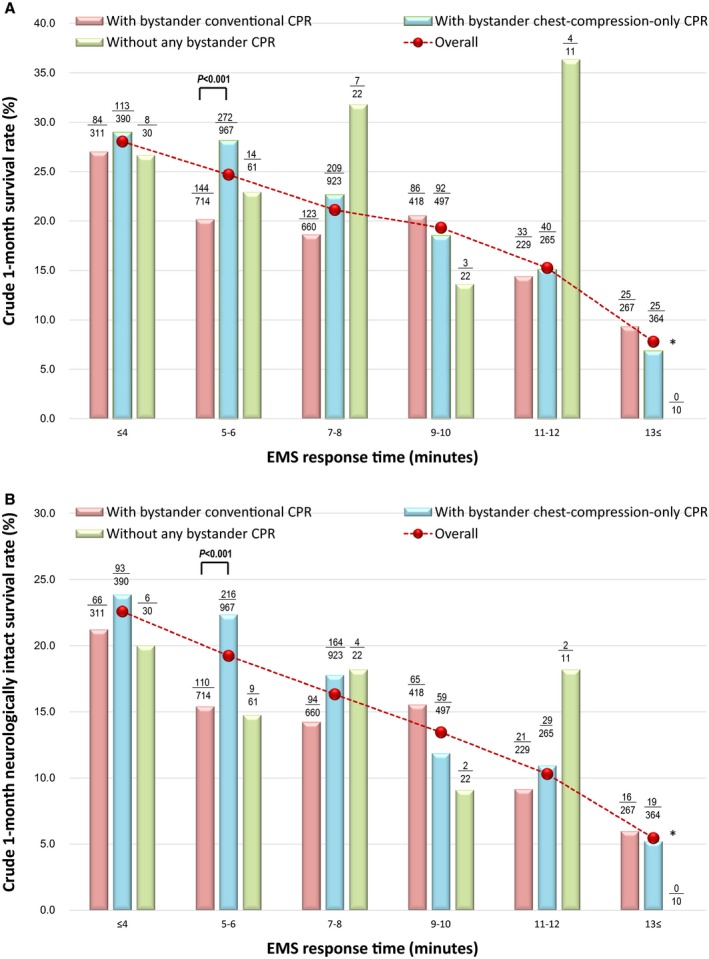
Crude 1‐month outcomes of 3 subgroups of the bystander defibrillation cohort by EMS response time. A, Crude 1‐month survival rate. B, Crude 1‐month neurologically intact survival rate. CPR indicates cardiopulmonary resuscitation; EMS, emergency medical services. *Statistical analysis was not available.

**Figure 8:**
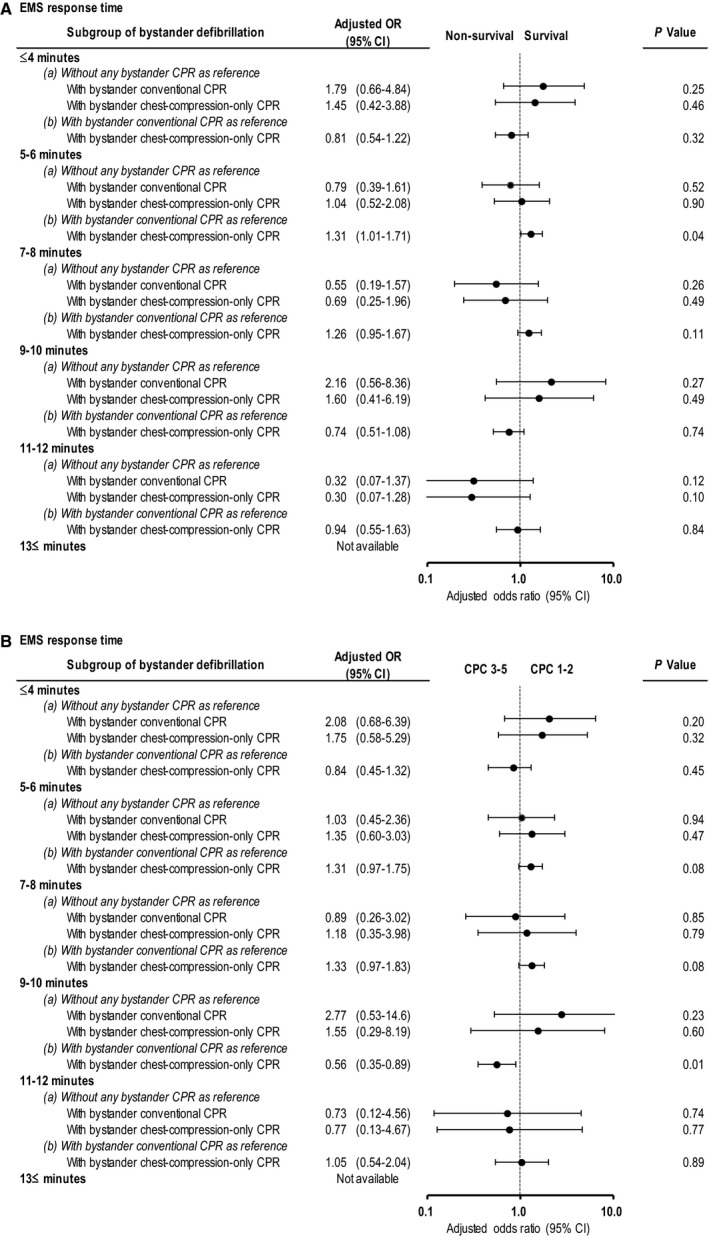
Adjusted ORs* of 3 subgroups of the bystander defibrillation cohort for 1‐month outcomes by EMS response time. A, 1‐month survival. B, 1‐month neurologically intact survival. CI indicates confidence interval; CPC, Cerebral Performance Category scale; CPR, cardiopulmonary resuscitation; EMS, emergency medical services; OR, odds ratio. *Adjusted ORs were calculated using a predefined set of 8 potential confounders: age, sex, witnessed arrest, initial shockable rhythm, cardiac cause, automated external defibrillator administration by EMS personnel, use of advanced airway management, and epinephrine administration.

Tables 3 and 4 show the results of subgroup analyses of overall 1‐month outcomes in patients who received bystander defibrillation. There were no significant differences in 1‐month outcomes among the 3 subgroups after adjusting for confounders. Figure 7A and 7B depict the crude 1‐month outcomes with EMS response time in the 3 subgroups of the patients who received bystander defibrillation. At 5 to 6 minutes of EMS response time, the crude rates of 1‐month survival and 1‐month neurologically intact survival were significantly lower among those who received bystander defibrillation and conventional CPR than those who received bystander defibrillation and chest‐compression‐only CPR. The multivariate logistic regression model revealed that, compared with bystander defibrillation with conventional CPR, bystander defibrillation with bystander chest‐compression‐only CPR was associated with increased odds for 1‐month survival at 5 to 6 minutes of EMS response time (adjusted OR, 1.31 [95% CI, 1.01–1.71], *P*=0.04), and decreased odds for 1‐month neurologically intact survival at 9 to 10 minutes of EMS response time (adjusted OR, 0.56 [95% CI, 0.35–0.89], *P*=0.01). These relationships of EMS response times and bystander defibrillation subgroups (ie, with conventional or chest‐compression‐only CPR or no CPR) with survival are depicted in Figure 8A and 8B in Forest plots. However, there were no significant differences in crude and adjusted 1‐month outcomes between bystander defibrillation without any bystander CPR and the other 2 subgroups by EMS response time.

**Table 3:** Overall 1‐Month Survival Rate of 3 Subgroups of the Bystander Defibrillation Cohort

	Bystander Defibrillation, n=6161		
	With Bystander Conventional CPR, n=2599, 42.2%	With Bystander Chest‐Compression‐Only CPR, n=3406, 55.3%	Without Any Bystander CPR, n=156, 2.5%
Crude rate (%)	495 (19.0)	751 (22.0)	36 (23.0)
Unadjusted OR (95% CI)	0.78 (0.53–1.15)	0.94 (0.64–1.38)	Reference
	Reference	1.20 (1.06–1.37)	1.27 (0.87–1.87)
Adjusted OR^a^ (95% CI)	0.98 (0.63–1.52)	1.03 (0.67–1.59)	Reference
	Reference	1.05 (0.91–1.22)	1.02 (0.66–1.58)

Values are reported as n (%) unless indicated otherwise. CI indicates confidence interval; CPR, cardiopulmonary resuscitation; EMS, emergency medical services; OR, odds ratio.

a Adjusted ORs were calculated using potential 9 confounders: age, sex, cause of cardiac arrest, initial rhythm, bystander witness status, prehospital epinephrine administration, defibrillation by EMS personnel, use of advanced airway management, and EMS response time.

**Table 4:** Overall 1‐Month Neurologically Intact Survival Rate of 3 Subgroups of the Bystander Defibrillation Cohort

	Bystander Defibrillation, n=6161		
	With Bystander Conventional CPR, n=2599, 42.2%	With Bystander Chest‐Compression‐Only CPR, n=3406, 55.3%	Without Any Bystander CPR, n=156, 2.5%
Crude rate (%)	372 (14.3)	580 (17.0)	23 (14.7)
Unadjusted OR (95% CI)	0.96 (0.61–1.52)	1.18 (0.75–1.86)	Reference
	Reference	1.23 (1.07–1.42)	1.04 (0.66–1.63)
Adjusted OR^a^ (95% CI)	1.29 (0.76–2.18)	1.37 (0.82–2.30)	Reference
	Reference	1.06 (0.90–1.25)	0.77 (0.46–1.30)

Values are reported as n (%) unless indicated otherwise. CI indicates confidence interval; CPR, cardiopulmonary resuscitation; EMS, emergency medical services personnel; OR, odds ratio.

a Adjusted ORs were calculated using potential 9 confounders: age, sex, cause of cardiac arrest, initial rhythm, bystander witness status, prehospital epinephrine administration, defibrillation by EMS personnel, use of advanced airway management, and EMS response time.

**Figure 9:**
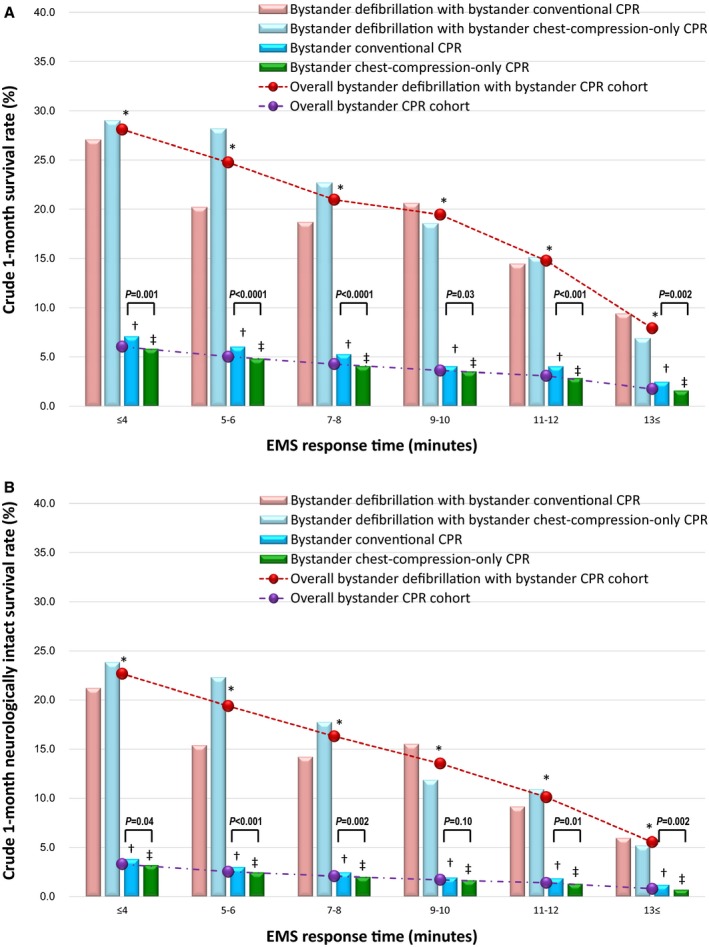
Crude 1‐month outcomes of 2 subgroups of the bystander defibrillation cohort and with 2 bystander CPR cohorts by EMS response time. A, Crude 1‐month survival rate. B, Crude 1‐month neurologically intact survival rate. CPR indicates cardiopulmonary resuscitation; EMS, emergency medical services. *P<0.0001; overall bystander defibrillation with bystander CPR cohort vs overall bystander CPR cohort. ^†^ *P*<0.0001; bystander defibrillation with bystander conventional CPR vs bystander conventional CPR. ^‡^ *P*<0.0001; bystander defibrillation with chest‐compression‐only CPR vs bystander chest‐compression‐only CPR.

**Figure 10:**
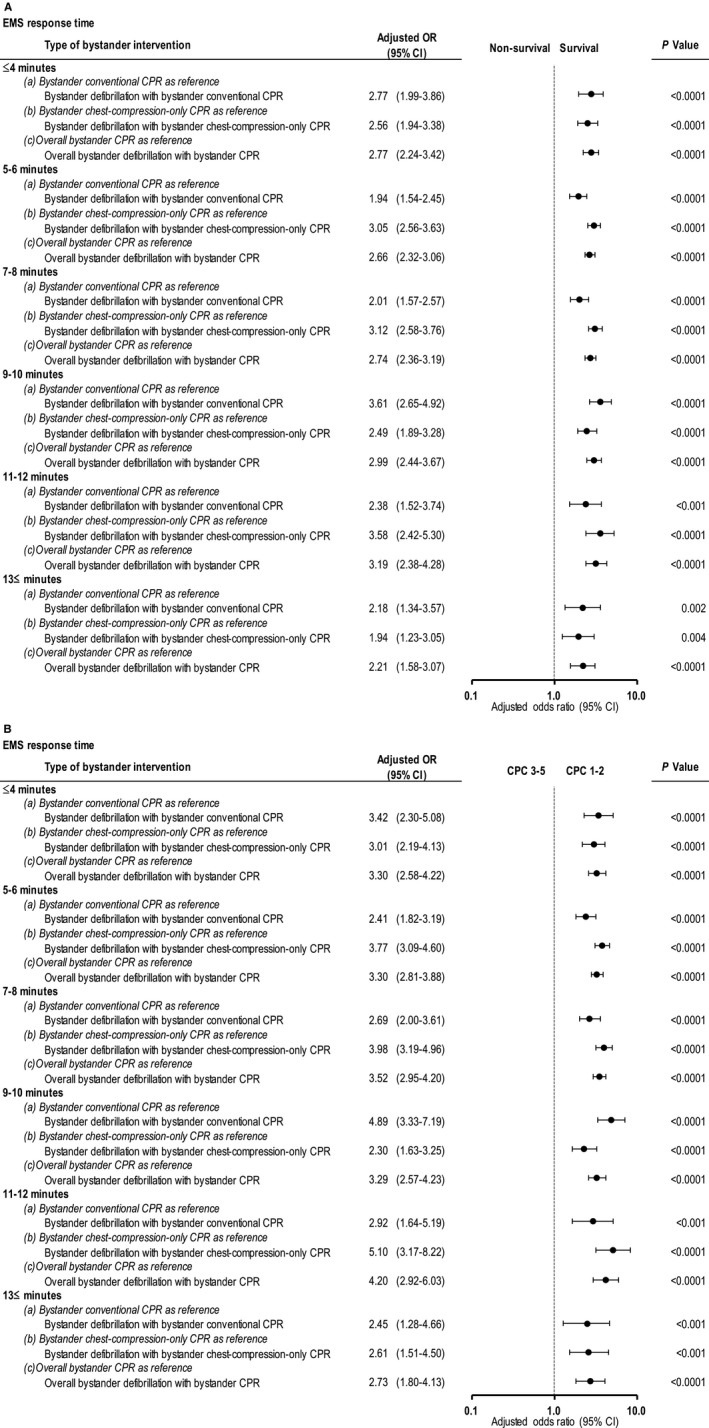
Adjusted ORs* of 2 subgroups of the bystander defibrillation cohort and the entire bystander defibrillation with bystander CPR cohort for 1‐month outcomes by EMS response time. A, 1‐month survival. B, 1‐month neurologically intact survival. CI indicates confidence interval; CPC, Cerebral Performance Category scale; CPR, cardiopulmonary resuscitation; EMS, emergency medical services; OR, odds ratio. *Adjusted ORs were calculated using a predefined set of 8 potential confounders: age, sex, witnessed arrest, initial shockable rhythm, cardiac cause, automated external defibrillator administration by EMS personnel, use of advanced airway management, and epinephrine administration.

Comparison of crude 1‐month outcomes (survival and neurologically intact survival) are graphed for each subgroup of those who received bystander defibrillation in Figure 9A and 9B, depicting survival at different EMS response times. Overall survival among the group receiving defibrillation with CPR is depicted in contrast to survival of those who received CPR without defibrillation. The rates of 1‐month survival and 1‐month neurologically intact survival associated with bystander defibrillation with bystander CPR (conventional CPR or chest‐compression‐only CPR) were significantly higher than those associated with bystander CPR alone (conventional CPR or chest‐compression‐only CPR) at every 2 minutes of EMS response time, respectively (all *P*<0.0001). The rates of 1‐month survival and 1‐month neurologically intact survival associated with bystander defibrillation with conventional CPR were significantly higher than those associated with bystander conventional CPR for every 2 minutes of EMS response time (all *P*<0.0001). The same results were observed in the comparison of bystander defibrillation with chest‐compression‐only CPR versus bystander chest‐compression‐only CPR (all *P*<0.0001). These findings were confirmed when adjusting for confounders, as depicted in Forest plots in Figure 10A and 10B.

## Discussion

This 5‐year, prospective, observational study from a Japanese OHCA registry documents an independent association between increases in EMS response time and decreased 1‐month survival and 1‐month neurologically intact survival, after adjusting for prehospital covariates. In particular, a 1‐minute increase in the EMS response time was associated with worse 1‐month survival and 1‐month neurologically intact survival by 9.3% (95% CI, 8.9–9.8%) and 10.7% (95% CI, 10.0–11.4%), respectively. The study also demonstrated that the upper limits of the EMS response times associated with increased 1‐month outcomes for any bystander intervention were as follows: (1) 13 minutes when bystanders provided defibrillation or conventional CPR and 11 minutes when bystanders provided chest‐compression‐only CPR, for 1‐month survival; and (2) 13 minutes when bystanders provided defibrillation and 11 minutes when bystanders provided CPR without defibrillation, for 1‐month neurologically intact survival. To the best of our knowledge, the present study is the first nationwide study to demonstrate the upper limit of EMS response time for which bystander interventions will still improve survival. The study's large sample size allowed for a more robust multivariate analysis of the correlates with neurologically intact survival and the precise estimates of ORs, according to EMS response times.

In the bystander defibrillation cohort, the vast majority of the patients received bystander CPR (conventional CPR or chest‐compression‐only CPR) in addition to bystander defibrillation (n=6005, 97.5%, Figure 1). Among the 3 subgroups of the bystander defibrillation cohort, no significant differences were found in overall 1‐month outcomes (ie, 1‐month survival and 1‐month neurologically intact survival) after adjusting for confounders (Tables 3 and 4). However, bystander defibrillation with chest‐compression‐only CPR was associated with decreased 1‐month neurologically intact survival at 9 to 10 minutes of EMS response time (Figure 8B). These results suggest that bystander defibrillation with conventional CPR may be preferable to bystander defibrillation with chest‐compression‐only CPR when EMS response time is long. Moreover, we found that the 1‐month neurologically intact survival rates associated with bystander defibrillation with bystander CPR (conventional CPR or chest‐compression‐only‐CPR) were significantly higher than those associated with bystander CPR alone, even with a long EMS response time (Figures 9B and 10B). These findings suggest that bystander defibrillation with bystander CPR may well be more effective than bystander CPR alone, considering the association with prolonged EMS response time.

EMS response time is an important determinant of survival after an OHCA.^16^, ^17^, ^18^ Several recent studies of OHCA have reported a negative effect of increasing EMS response time on clinical outcomes, a finding consistent with our study.^10^, ^19^, ^20^, ^21^, ^22^, ^23^ Rajan et al^10^ reported that the absolute increase in 30‐day survival associated with bystander CPR (bystander defibrillation not included) decreased rapidly with increased EMS response time, and that the association between 30‐day survival and bystander CPR, compared with the absence of bystander CPR, became statistically insignificant when the EMS response time exceeded 13 minutes: 3.7% (95% CI, 2.2–5.4) for bystander CPR versus 1.5% (95% CI, 0.6–2.7) for no bystander CPR. These results, too, are consistent with our study. Our study clarified the association between 1‐month neurologically intact survival and bystander interventions at different EMS response times.

To increase the rates of bystander intervention before the arrival of EMS personnel, several strategies have been implemented in industrialized nations: traditional classroom training, dispatcher‐guided CPR, school‐based instruction, online education, and targeted neighborhood or individualized training.^4^, ^7^, ^8^, ^9^, ^24^, ^25^, ^26^, ^27^, ^28^, ^29^, ^30^ Despite these strategies and the increase in rates of bystander intervention, the rate of neurologically intact survival cannot improve if the EMS response time is too long. In the present study, 8.0% (495/6161) of the patients who received bystander defibrillation had an EMS response time of >13 minutes, and 12.5% (32 246/257 116) of those who received any bystander CPR had an EMS response time of >11 minutes. Despite these bystander interventions, the outcome of this population (12.4%; 32 741/263 277) was not significantly better than the outcome of those who received no bystander intervention. Therefore, appropriate initiatives should be adopted to reduce EMS response time.^31^, ^32^, ^33^ Such initiatives may include the reconfiguration of emergency call systems to enable rapid arrival of lay‐trained volunteers before the arrival of EMS personnel at the scene, or the implementation of educational programs to improve performance of high‐quality bystander CPR with defibrillation.^7^, ^30^, ^34^, ^35^, ^36^, ^37^, ^38^, ^39^, ^40^, ^41^, ^42^

We defined EMS response time as the time from the call receipt by an EMS center to the arrival of an ambulance at the site of an OHCA. Therefore, the EMS response time included the following: call receipt to EMS notification (activation interval), EMS notification to vehicle wheels rolling (turnout interval), and EMS wheels rolling to arrival at the scene (travel interval).^32^, ^33^ As the activation and turnout intervals are reportedly around 1 minute,^31^, ^32^, ^33^ a longer response time may be predominantly attributed to the travel interval. The distribution of EMS centers in the different regions of Japan is approximately proportional to the population densities.^12^ Therefore, while longer EMS response times in low population density areas (rural areas) in Japan could be attributed to the distance between ambulances and patients with OHCA,^19^ in high population density areas, they could be attributed to increases in the incidences of traffic congestion and number of building construction sites.^33^ Silverman and colleagues^43^ reported that the time difference between arrival at the location and arrival at the patient's side is an important component of overall EMS response time in large urban areas, particularly in multistory buildings. In the present study, however, we could not analyze this time interval, sometimes referred to as “vertical response time,” because of lack of data. During the 5 years of our study, the EMS response time significantly increased, from 7.67 minutes (95% CI, 7.66–7.70) in 2010, to 7.96 minutes (95% CI, 7.94–7.99) in 2014 (*P*<0.001). This may be partially explained by the increase in the dispatched number of ambulances (6.0% increase for 5 years; 1015 times per EMS personnel per year in 2010 versus 1076 per EMS personnel per year in 2014).^12^ Considering these factors, improving the travel interval may be difficult in Japan without changes to EMS responder deployment (both in numbers or locations). Nichol et al^31^ found that a 30‐s decrease in the activation interval was associated with a 0.7% increase in survival to discharge, and postulated that improving the activation interval may be a viable approach to improving the outcomes of OHCA. To reduce the activation interval, Cone et al^34^ suggested (1) reconfiguring the communication systems to eliminate the need to transfer an EMS call from a primary public safety answering point to a secondary public safety answering point and (2) providing regular, sustained feedback to the dispatchers, with regard to the activation interval for OHCA, as part of quality management programs.^35^ Unlike for field providers, the quality management process for EMS dispatchers includes a qualification of protocol compliance, obtained via monitoring recordings of EMS calls.^35^ To reduce the time between the EMS dispatcher's identification of possible cardiac arrest and the initiation of EMS‐directed bystander CPR, more information regarding time‐based metrics would be required, such as the time interval between the received call and the first chest compressions performed by the caller.^44^ The Japanese FDMA has developed a training program for dispatchers to improve their communication, diagnosis, and instructional skills.^45^ However, a reduced time to the initiation of EMS‐directed bystander CPR has not yet been shown.

In the present study, the EMS response time in the cohort that received no bystander intervention was significantly shorter than that in the other 3 cohorts (Table 1). It is possible that the dispatcher could not direct the layperson fully in performing bystander interventions before the ambulance arrived, because the OHCA occurred very close to the location of the responding ambulance. However, we did not have sufficient data to clarify this observation.

Initiatives to improve survival from OHCA should also focus on factors other than the EMS response time that may influence survival. Such initiatives may include first responder systems for early defibrillation and CPR such as police CPR‐AED programs,^36^, ^37^ the HeartRescue Project in North Carolina,^7^ text message alert systems,^38^ mobile‐phone positioning systems to dispatch lay volunteers who are trained in CPR,^30^ mobile application–based alert systems for trained responders,^39^ the PulsePoint Respond mobile device application for the recruitment of citizens to perform basic life support,^40^ and delivering defibrillators using a drone.^41^ In Japan, a rural community first responder system involves nonmedical citizens trained in resuscitation.^42^ In Tokyo, a system that uses social media is in place to notify lay volunteers of nearby cardiac arrests.^34^ However, the efficiency of these initiatives in recruiting first responders or lay responders has not yet been demonstrated.

There is no evidence that high‐quality bystander CPR training can compensate for longer EMS response times. However, delivery of higher‐quality bystander‐initiated CPR before the arrival of EMS personnel may help to compensate for longer EMS response times. Although the prognosis of elderly patients with OHCA has significantly improved in Japan,^46^ it could be hypothesized that, especially in rural areas, elderly people with episodes of OHCA may be more likely to receive CPR from elderly bystanders; therefore, it is suggested that the quality of the administered CPR may be poor, resulting in lower neurologically intact survival rates.^42^ In 2015, local fire departments in Japan trained ≈1.4 million citizens through conventional 3‐hour CPR programs, consisting of chest compressions, mouth‐to‐mouth ventilation, and AED.^12^ The dissemination of high‐quality bystander CPR training programs for elderly individuals is key in compensating for longer EMS response times and may lead to an increase in the upper limit of the EMS response times associated with good outcomes after bystander intervention.

The present study has some potential limitations. First, the different job roles and experience levels of the EMS providers might influence the EMS response times. However, we could not analyze the differences among the EMS providers because of lack of sufficient data. Second, we cannot exclude the possibility of uncontrolled confounders, although we used a uniform data‐collection procedure as well as a large sample size and a population‐based design. We lacked data on items such as pre‐existing comorbidities, the location of the arrest, the quality of bystander CPR, the exact amount of time taken for the initiation of bystander CPR and defibrillation after OHCA, the quality of EMS CPR during transport, the EMS scene time, and in‐hospital treatments (eg, postresuscitation targeted temperature management). Therefore, we could not include these data in our analyses. Moreover, increasing the provision of bystander CPR in the bystander defibrillation cohort could contribute to increased survival. Third, as with all epidemiological studies, an ascertainment bias as well as a lack of integrity and validity of the data act as potential limitations. Fourth, the relevance of our results to other communities, which have different emergency care systems and protocols, remains unknown. Therefore, similar studies in other countries may be required to validate our results.

## Conclusions

EMS response time was found to be independently associated with decreased 1‐month neurologically intact survival after OHCA. The upper limits of the EMS response times associated with improved 1‐month neurologically intact survival were 13 minutes when bystanders provided defibrillation and 11 minutes when bystanders provided CPR without defibrillation.

## Sources of Funding

This work was supported by the Japan Society for the Promotion of Science (KAKENHI Grant Number 15K08543), which had no role in the design and implementation of the study, the analysis and interpretation of the data, or the approval of the article.

## Disclosures

None.
